# High-resolution isotope dietary analysis of Mesolithic and Neolithic humans from Franchthi Cave, Greece

**DOI:** 10.1371/journal.pone.0310834

**Published:** 2025-01-17

**Authors:** Valentina Martinoia, Anastasia Papathanasiou, Sahra Talamo, Rebecca MacDonald, Michael P. Richards

**Affiliations:** 1 Department of Archaeology, Simon Fraser University, Burnaby, B.C., Canada; 2 Ephorate of Paleoanthropology and Speleology, Greek Ministry of Culture, Athens, Greece; 3 Department of Chemistry G. Ciamician, Alma Mater Studiorum, University of Bologna, Bologna, Italy; University of California Santa Cruz, UNITED STATES OF AMERICA

## Abstract

Franchthi Cave, in the Greek Peloponnese, is a well-known Paleolithic, Mesolithic and Neolithic site, with several human burials. In many parts of Europe there is clear evidence from archaeological and isotopic studies for a diet change between the Mesolithic and Neolithic periods. This is especially the case in coastal contexts where there is often a shift from predominantly marine food diets in the Mesolithic to terrestrial (presumably domesticated) foods in the Neolithic. However, at Franchthi Cave previous isotope research did not show changes in diets between these two periods, and also showed relatively little input from marine foods in diets in either time period, despite the coastal location of the site and the presence of marine shellfish and fish, including tuna. High-resolution compound specific amino acid isotope analysis reported here from humans from the Lower Mesolithic and Middle Neolithic periods confirms the previous bulk isotope results in showing little or no consumption of marine foods in either time period. However, it is important to note that our isotopic sample does not come from episodes when tuna is abundant and therefore do not cover the whole range of known diets from the site. Conversely, in our sample there is some evidence of marine food consumption (likely seaweed) by sheep in the Neolithic period. We also report here five direct AMS radiocarbon dates for the five analyzed humans from the site.

## Introduction

**Fig 1:**
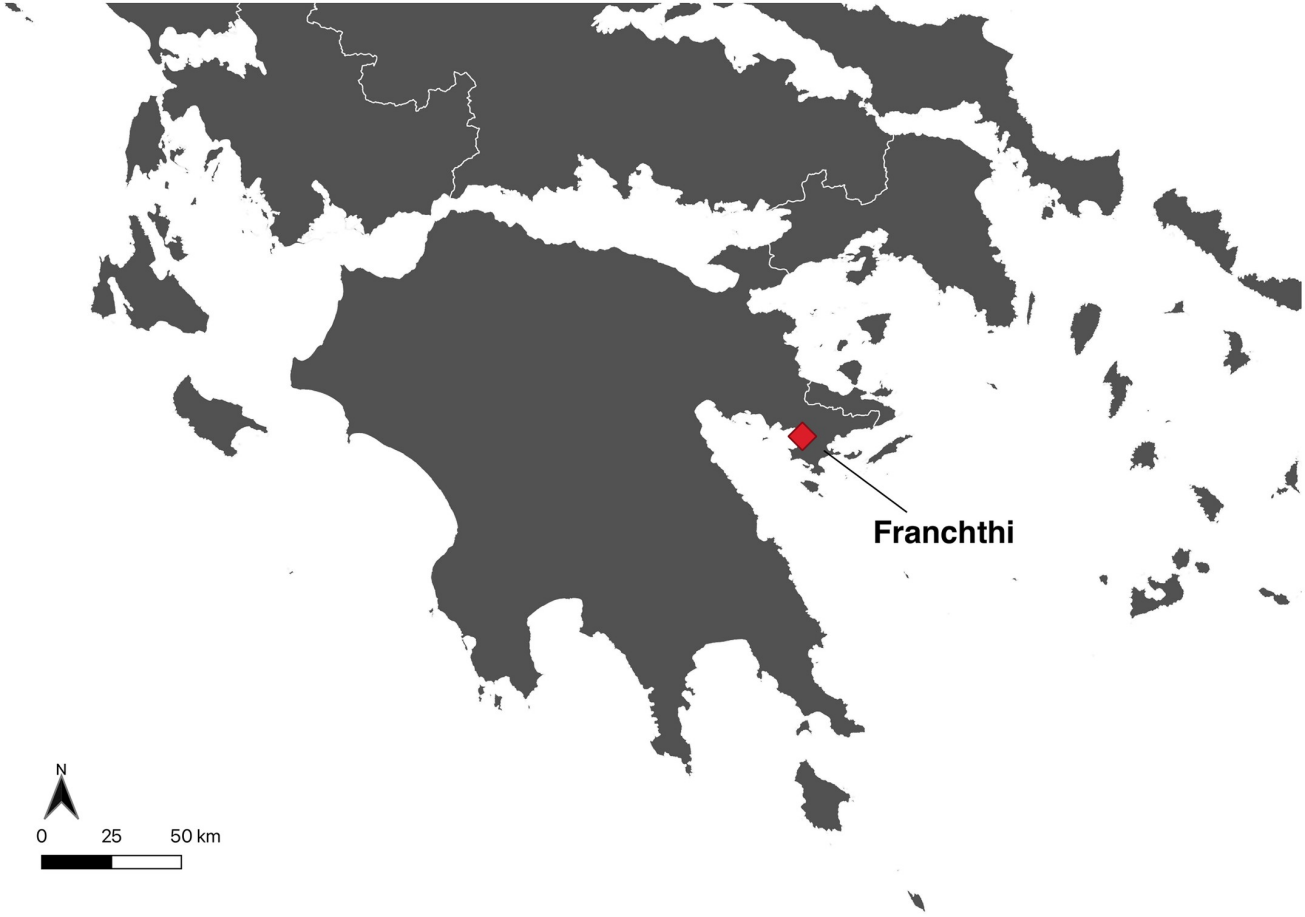
Geographical location of Franchthi Cave.

Franchthi Cave is today located on the southwestern shore of the Argolid peninsula of the Peloponnese, overlooking the Bay of Koilada (Fig 1). Thomas W. Jacobsen (Indiana University) directed the excavation of the cave between 1967 and 1979 in collaboration with the University of Pennsylvania and the American School of Classical Studies at Athens [1]. There are nearly 40,000 years of stratigraphic sequence in the cave, from the earliest Upper Paleolithic through the Final Neolithic [2–6]. In the Greek peninsula, few other sites have produced a stratigraphic sequence for prehistory as continuous as Franchthi (e.g., Klisoura cave in the Argolid and Theopetra in Thessaly) [7, 8]. Franchthi is therefore a crucial site to advance our understanding of pre-Neolithic Greece specifically, and of the Mesolithic—Neolithic transition in Europe more generally.

In fact, the origins and development of agriculture marked the transition from the Mesolithic to the Neolithic, thus constituting a pivotal turning point in the prehistory of Europe [9]. The Mesolithic—Neolithic transition was accompanied by significant changes in human lifestyle and greatly influenced human subsistence strategies [10], leading to the development of new agropastoral economies all over Europe. Furthermore, isotopic evidence has shown that dietary patterns changed significantly with the onset of the Neolithic period across Europe [9, 11]. As the Neolithic began, most coastal European communities have been shown to have shifted from the typical Mesolithic marine-based diet to one dominated almost exclusively by terrestrial resources with the arrival of the “Neolithic package” [9, 11–17].

In contrast, some research [18, 19] suggests that coastal Mesolithic hunter-gatherers in the Mediterranean may have not relied significantly, if at all, on the consumption of aquatic resources for sustenance. As a matter of fact, the dietary adaptations of Mesolithic communities in the Mediterranean are often assumed to have been different from the maritime-focused hunter-gatherer economies that developed along the Atlantic and Baltic coasts [18]. This is because of the Mediterranean’s peculiar biogeographical qualities (e.g., low intertidal area and primary productivity), which are considered to be the cause for a general lack or insufficiency of aquatic food resources to sustain human populations, especially during the Holocene [20]. Nonetheless, marine mammals, fish and mollusc remains can still be found in most zooarchaeological assemblages from Mediterranean Mesolithic coastal sites, indicating that, at the very least, the regular terrestrial-based diet was supplemented by the occasional intake of aquatic resources [18].

Specifically in the Aegean, it has been noted [21, 22] that prehistoric fishing strategies were characterized by two distinct exploitation patterns. The first one was focused on year-round coastal and nearshore fishing and shellfish gathering activities and was practiced from the Mesolithic throughout the entire Neolithic. Conversely, the second type of marine exploitation pattern, observed at sites like Cyclops cave in the Sporades [23–27], appears to have been more focused toward seasonal fishing of pelagic resources like tunas and mackerels (even though all coastal resources still remained heavily exploited in the early phases of the Mesolithic). This latter pattern is considered to have been generally confined within the Mesolithic period and to not have continued in the Neolithic [21]. The occupants of Franchthi seem to have practiced the first pattern of marine resources exploitation in the early phases of the Mesolithic (characterized by a significant presence of marine shell remains) and shifted towards the second during the Upper Mesolithic (when remains of bluefin tuna dominate the faunal assemblages). However, the exploitation of sea resources at Franchthi did not stop with the beginning of the Neolithic.

During the Lower Mesolithic (LM), which dates to the 10^th^ millennium BP in correspondence with the euthermic climatic phase of the Preboreal [28, 29], Franchthi Cave was located up to 2 kilometers from the coast, above a grassy coastal plain divided by a river [30, 31]. The palaeobotanical assemblage during this period is characterized by a remarkable amount (~28,000) of seeds—predominantly from fruits (e.g., pistachios [*Pistacia* sp.], pears [*Pyrus amygdaliformis*], and almonds [*Prunus dulcis*]), wild legumes (e.g., lentils [*Lens culinaris*]), and wild cereals (e.g., oats [*Avena sativa*] and barley [*Hordeum vulgare*]) [28, 32, 33]. The LM zooarchaeological assemblage reveals the preferential hunting of deer (*Cervus elaphus*) and wild boar (*Sus scrofa*), while the remains of aurochs *(Bos primigenius*), fox (*Vulpes vulpes*), and hare (*Lepus europaeus*) see a slight reduction compared to previous periods [19]. Conversely, a substantial number of land snails (*Helix figulina*)—totaling roughly 55,000 shells—were also recovered from LM layers in just two of the four excavated trenches and were likely a consistent part of the human diet [28].

Additionally, the number of marine shells, collected year-round [34], sees a dramatic increase in the LM (~16,000 shells) in comparison to the previous Final Paleolithic period (~1,800 shells) [30]. While there has been some debate around their consumption [see: 28], it is now widely accepted [28, 35] that certain species of marine molluscs served an exclusively ornamental function and were not consumed during this period with only species like *Patella*, *Hexaplex*, and *Cerastoderma* being likely consumed as food, although sporadically. Perlès notes further that this infrequent consumption of molluscs is in line with the small amount of shallow-water fish bones (e.g., eels [*Conger conger*], sea breams [Sparid], and mullets [*Mugil cephalus*]) recovered from the LM layers at Franchthi, overall pointing towards a reduced exploitation of marine resources compared to some earlier phases.

There is therefore no doubt that this combination of natural shelter and abundant resources in a favorable climatic condition played an important role in supporting human activity at Franchthi Cave during the LM. As for the nature of this activity, the presence of ten human burials (incomplete remains of eight interments and two cremations) and several more individuals represented in bone scatter in the LM layers attest to the use of the cave for disposal of the dead as well as a place for the living [36]. Perlès [28] goes so far as to suggest that during this period the cave was used specifically as a burial ground, rather than serving as a seasonal or year-round habitation center for hunter-gatherer groups. Specifically, the hypothesis of Franchthi being visited repeatedly and at all times of year as a “place for the dead” [28 p123] during the LM would be supported by the elevated number of seed remains (possibly consumed in the context of ritual feasting) and perforated sea shells (used as ornaments)–which strongly suggests (especially by Perlès) the undertaking of ritualistic or ceremonial activities [28, 37], although this evidence does not negate the possibility that the cave may have been used as a place of occupation and other activities “by the living” as well.

The Upper Mesolithic period at Franchthi saw a marked increase in the exploitation of marine resources. Specifically, tuna remains dominate the fish assemblage from this period [19, 33, 38], underscoring the intensification of open and deepwater fishing practices and the importance of tuna as a key dietary component for the Franchthi foragers. Unfortunately, this study did not include the analysis of human remains from the Upper Mesolithic phase.

The onset of the Neolithic with agriculture and food production being the predominant economic strategy at Franchthi is accompanied by some changes: the use of the site expands from the cave to a sector which is today located on the beach (Paralia), although it is not clear if during the Final Neolithic this area was used for habitation or ritual purposes [39]. Fishing activities experience a significant decline during the Initial Neolithic period (8650–8450 cal. BP), followed by an increase during the Early (8450–7800 cal. BP) and Middle Neolithic (7800-7300cal. BP) [40]. Domestic species (e.g., ovicaprids [*Ovis aries* and *Capra hircus*], cattle [*Bos taurus]*, and pigs [*Sus scrofa*]) dominate the assemblages throughout the entire Neolithic, while small game (i.e., fish, birds, and mammals) is present in higher percentages in the Early and Middle Neolithic compared to the subsequent Early Late Neolithic 1 period [40]. The Middle Neolithic, specifically, saw an increase in the importance of both pigs and fish. This, in association with a more intense use of the Paralia and the abundance of ceramic remains, suggests that frequentation of the settlement peaked at this time [40].

After the Middle Neolithic, fish levels at Franchthi see some fluctuations. During the Early Late Neolithic (ELN1; 7300–6800 cal. BP), the number of fish remains drop to <1% of total faunal remains, but notably increases again during the ELN2 (6800–6500) [41, 42], when “fish increases to 20–40% of the total bulk of animal bones” [3 p66]. Lastly, during the Final Neolithic (6500–5700 cal. BP), fishing activities decrease once more [40].

Previous isotope studies on the human and faunal remains from Franchthi Cave [43–45] suggested that during both the Mesolithic and the Neolithic, the occupants of this site consumed a varied diet based exclusively on terrestrial resources [45]. Similarly, none of the individuals from Mesolithic—Neolithic Franchthi previously analyzed for bulk collagen stable isotope analysis (SIA) seemed to show δ^15^N values consistent with a more than minor consumption of marine resources, despite the proximity of this site to the Mediterranean waters and the zooarchaeological record recovered from the Mesolithic layers [45].

Given these premises, it becomes clear that a deeper and more detailed understanding of the subsistence strategies of the individuals who occupied Franchthi during the Mesolithic and the Neolithic periods is needed. In this paper we therefore present new bulk collagen and compound-specific stable isotope data, as well as new radiocarbon dates, to investigate human diet at Franchthi Cave during the Lower Mesolithic and Middle Neolithic periods and to add to our current knowledge of human subsistence strategies in the coastal Mediterranean during the Holocene.

CSIA-AA offers the potential to overcome some of the limitations posed by bulk tissue stable isotope analysis (SIA) in the study of paleodiets (e.g., source equifinality, dietary complexity, baseline isotopic variability, and metabolic isotopic fractionation), providing a more detailed understanding and interpretation of trophic levels and resource consumption than bulk SIA [46, 47]. The significance of the application of this technique to Franchthi Cave is twofold. On the site-specific level, Franchthi Cave offers an ideal context to investigate human subsistence patterns since it presents a continuous stratigraphy spanning pivotal periods in human prehistory, such as the Meso-Neolithic transition. This is particularly important in Greece as this is the first area in Europe where the Neolithic began [6, 29, 48]. On a broader scale, the use of this technique contributes to further our understanding of the variability of the dietary adaptations of Mesolithic and Neolithic populations in the Mediterranean region.

## Material

The material is kept at the Nafplion Archaeological Museum (Greece). All necessary permits were obtained for the described study, which complied with all relevant regulations. The permits to analyze the remains were issued by the Greek Ministry of Culture to M. Richards. A total of 49 human and faunal bone fragments from the Mesolithic and Neolithic layers at Franchthi were analyzed for bulk collagen stable isotope analysis (SIA) at the Max Plank Institute for Evolutionary Anthropology (S7 Table). Of these, five human and six faunal remains produced enough collagen to be used for compound-specific isotope analysis of individual amino acids (CSIA-AA) (Table 1) and were therefore sent to the Isotope Laboratory, Department of Archaeology, Simon Fraser University (Canada), where they were reanalyzed for δ^13^C, δ^15^N, and δ^34^S bulk collagen SIA and analyzed for CSIA-AA. The five humans selected for CSIA-AA were also pretreated for radiocarbon analysis at the BRAVHO Laboratory (University of Bologna, Italy) and analyzed for ^14^C at the Curt-Engelhorn Center for Archaeometry in Mannheim (Germany).

**Table 1:** List of samples selected for CSIA-AA.

SSFU	S-EVA	Original #	Species	Age	Period
2705	4145	Fr2 (FRA-101)	Human	30–35 years	Lower Mesolithic
2706	4148	Fr6 (FRA-104)	Human	6–8 months	Lower Mesolithic
2707	4150	Fr11 (FRA-106)	Human	4–5 years	Early Middle Neolithic
2708	4154	Fr37 (FRA-110)	Human	4–5 years	Middle Neolithic
2709	4155	Fr12 (FRA-111)	Human	8 years	Early Middle Neolithic
2710	4113	FRA-6	Canid	ND	Mesolithic
2711	4128	FRA-21	Canid	ND	Mesolithic
2713	4138	FRA-31	Canid	ND	Neolithic
2714	4140	FRA-33	Pig	ND	Neolithic
2715	4141	FRA-34	Sheep/goat	ND	(Middle?) Neolithic
2716	4142	FRA-35	Sheep/goat	ND	(Middle?) Neolithic

## Methods

### Bulk stable isotope analysis

For the 49 MP-EVA samples, the collagen was extracted following the protocol described by Müldner and Richards [49]. Specifically, 250–450 mg of bone were cleaned via mechanical abrasion and demineralized in 0.5 M HCl at 4°C for several days. Once demineralized, the samples were rinsed with distilled water and gelatinized at 75°C for 48 hours in a pH 3 HCl solution. After using 9ml Ezee filter separators for the removal of reflux-insoluble residues, the remainder of the solution was filtered through 30-kDa MWCO ultrafilters. The supernatant >30-kDa fraction was frozen for 24 hours and then lyophilized for δ^13^C and δ^15^N EA-IRMS (Elemental Analysis with Isotope Ratio Mass Spectrometry) analysis.

### Radiocarbon analysis

The selected five human samples were prepared for radiocarbon analysis following the pre-treatment protocol described by Talamo and Richards [50] and Talamo et al. [51]. In brief, samples were demineralised in HCl 0.5 M at 4°C until soft and CO_2_ effervescence had stopped, with HCl changed twice per week. The demineralised samples were treated with NaOH 0.1 M for 30 min to remove humic acid contamination and then re-acidified in HCl 0.5 M. Samples were then gelatinised in HCl pH3 at 75°C for 20 h before being filtered to remove particles >60–90 μm (Ezee filters, Elkay Labs, UK) and ultrafiltered to concentrate the >30 kDa fraction (Sartorius VivaSpin Turbo 15). Filters were precleaned prior to use [51, 52]. The >30 kDa fraction was lyophilised for 48 h and the collagen was immediately weighed to determine the collagen yield as a percentage of the dry sample weight.

All extracts were characteristic of well-preserved collagen [51] so were submitted for dating via Accelerator Mass Spectrometry (AMS). The extracts were sent to the Curt-Engelhorn Center for Archaeometry in Mannheim, Germany (CEZA, lab code: MAMS), where the collagen was combusted to CO_2_ in an elemental analyzer (EA) and converted catalytically to graphite, before being measured on a MICADAS AMS [53].

### CSIA-AA

Preparation of the samples for CSIA-AA involved hydrolysis and derivatization of the collagen amino acids as follows. 1 mg of lyophilized collagen was hydrolyzed in 6M HCl for 20 hours, evaporated under a gentle stream of nitrogen, and then placed in a heat block at 110°C for 24 hours. The samples were subsequently evaporated under a gentle stream of N_2_ at room temperature, dissolved in a weak acid and frozen overnight. Three internal (QC) collagen standards (SRM-1 [seal collagen], SRM-2 [deer collagen], SRM-3 [commercial bovine collagen]) were processed and analyzed together with the collagen samples.

The following control standards were prepared before proceeding with derivatization:

1. Two AA mixtures containing the following amino acids: alanine (Ala), glycine (Gly), valine (Val), leucine (Leu), threonine (Thr), serine (Ser), proline (Pro), aspartic acid (Asp), glutamic acid (Glu), hydroxyproline (Hyp), lysine (Lys), and phenylalanine (Phe). The δ^13^C and δ^15^N values of these AAs were previously analyzed using EA-IRMS, with the exception of Gly (USGS65) and Val (USGS74), for which certified values exist;
2. One QC mix containing Gly, Glu, Pro, and Val;
3. 150 μL of an internal norleucine (Nle) standard that was added to all samples and standards.

The derivatization of all the samples and standards was performed using N-acetyl isopropyl (NAIP) esters, as described by Corr and colleagues [54]. GC-C-IRMS analysis of the δ^13^C and δ^15^N values of the derivatized AAs was also performed at the Archaeology Isotope Laboratory, Department of Archaeology, Simon Fraser University (Canada).

For carbon CSIA-AA we used a VF-23ms column (Agilent), whose specs are 60 m x 0.32 mm x 0.15 μm film thickness. Prior to each run the reactor was oxidized for 60 minutes, followed by a backflush (BF) for 60 minutes. Each sample and standard were additionally subjected to a seed oxidation of 2 minutes followed by a BF of 2 minutes. Injection occurred at 250°C with a flow of 1.5 ml/min (injection volume is 1.1 μL). The oven program is 70°C for 0.5 minutes, increase at a rate of 15°C/min to 120°C, then increase at a rate of 2°C/min to 180°C, then increase at a rate of 5°C/min to 250°C, where it is held at this final temp for 21 minutes.

For nitrogen CSIA-AA we used a custom DB-35 column (Agilent), whose specs are 60 m x 0.32mm x 0.50 μm film thickness. Prior to each run the reactor was oxidized for 30 mins then BF for 60 minutes. Each sample and standard were additionally subjected to a seed oxidation of 0.2 min (12 seconds) then BF for 0.4 minutes (24 seconds). Injection occurred at 240°C with a flow rate of 1.3 ml/min, and the injection volume was also 1.1 μL. The oven program is 60°C for 2 minutes, then the temperature was increased at a rate of 15°C/min to 120°C, then increased at a rate of 2°C/min to 210°C where it was held for 1 minute. Then the temperature was increased again at a rate of 5°C/min to 280°C, where it was held for 18 minutes.

## Results and discussion

### Bulk stable isotope analysis

**Fig 2:**
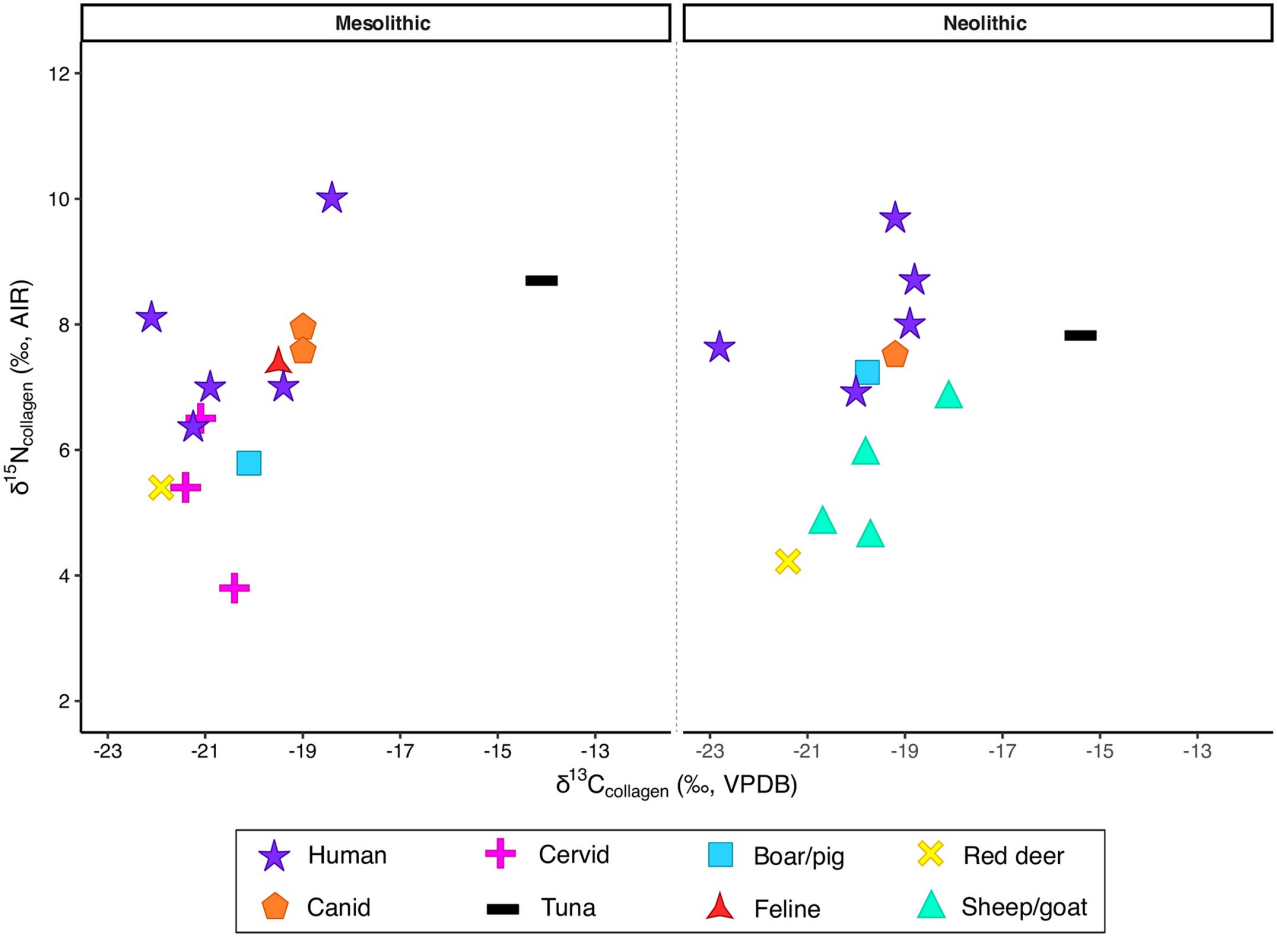
Bulk δ^13^C and δ^15^N results for the Mesolithic and Neolithic samples from Franchthi (S-EVA values).

Of the 49 original samples analyzed at MP-EVA, only 27 produced enough collagen for EA-IRMS analysis (Table 2 and Fig 2). Of these, 11 were reanalyzed at Simon Fraser University. Specifically, we selected only human and faunal samples that had a C:N ratio between 3.2 and 3.6, an extracted collagen mass of >2 mg and a %collagen as close as possible to 1 (in our case, >0.7) to ensure that enough material would be available after the SFU re-run for bulk tissue isotope analysis (which also included δ^34^S analysis). Since the values obtained at MP-EVA and those obtained at SFU are extremely similar, but not all 27 samples were reanalyzed at Simon Fraser University, in this section only the δ^13^C and δ^15^N bulk SIA results will be discussed, using the MP-EVA values for consistency purposes. At SFU, δ^34^S values were also calculated for the samples that presented enough collagen to perform sulfur SIA, specifically for three humans (SSFU 2705–2707), two ovicaprids (SSFU 2715 and 2716), and one canid (SSFU 2713). The δ^34^S values for these samples, are reported in Table 2 and are consistent with the location of the site near the coast [55, 56].

**Table 2:** List of the bulk δ^13^C and δ^15^N isotope analysis for Mesolithic and Neolithic humans and animals from Franchthi Cave.

Age	Species	Original #	S-EVA	SSFU	Element	Context	δ^13^C (MP-EVA)	δ^15^N (MP-EVA)	δ^13^C (SFU)	δ^15^N (SFU)	δ^34^S (SFU)
Mesolithic	Cervid	FRA-11	4118		long bone fragment	Trench G1	-21.4	5.4			
Mesolithic	Cervid	FRA-12	4119		metapodial	Trench G1	-20.4	3.8			
Mesolithic	Cervid	FRA-25	4132		1st phalanx	Trench G1	-21.1	6.5			
Mesolithic	Canid	FRA-6	4113	2710	mandible	Trench G1	-19.0	7.6	-19.0	7.6	
Mesolithic	Canid	FRA-21	4128	2711	mandible	Trench G1	-19.0	7.9	-19.0	7.9	
Mesolithic	Feline	FRA-7	4114		humerus	Trench G1	-19.5	7.4			
Lower Mesolithic	Human	Fr 1 (FRA-100)	4144		rib and long bone fragment	G1:60	-22.1	8.1			
Lower Mesolithic	Human	Fr 2 (FRA-101)	4145	2705	rib and long bone fragment	G1:65	-19.4	7.0	-19.6	7.3	11.6
Lower Mesolithic	Human	Fr 5 (FRA-102)	4146		long bone fragment	G1:65	-20.9	7.0			
Lower Mesolithic	Human	Fr 6 (FRA-104)	4148	2706	ribs	G1:65	-18.4	10.0	-18.6	10.2	10.8
Upper Mesolithic	Human	Fr 410 (FRA-105)	4149		iliac crest fragment	G1:31	-21.2	6.4			
Mesolithic	Pig	FRA-22	4129		mandible	Trench G1	-20.1	5.8			
Mesolithic	Red deer	FRA-23	4130		mandible	Trench G1	-21.9	5.4			
Mesolithic	Tuna	FRA-27	4134		vertebrae	Trench G1	-14.1	8.7			
Neolithic	Dog	FRA-31	4138	2713	ulna	FF1:41D	-19.2	7.5	-19.4	7.9	12.1
Early Middle Neolithic	Human	Fr 11 (FRA-106)	4150	2707	long bone fragment	A:62	-19.2	9.7	-19.3	10.0	12.3
Neolithic	Human	Fr 62 (FRA-107)	4151		long bone fragment	Q5NE:2-11Sk1	-22.8	7.6			
Middle Neolithic	Human	Fr 78 (FRA-109)	4153		hand bones	Q55:216	-18.9	8.0			
Middle Neolithic	Human	Fr 37 (FRA-110)	4154	2708	mandible	H:37K	-18.8	8.7	-18.8	8.7	
Middle Neolithic	Human	Fr 12 (FRA-111)	4155	2709	rib	FF1:41D	-20.0	6.9	-20.0	6.9	
Neolithic	Pig	FRA-33	4140	2714	mandible	FF1:41D	-19.8	7.2	-19.8	7.2	
Neolithic	Red deer	FRA-30	4137		metatarsal	FF1:41D	-21.4	4.2			
Neolithic	Sheep/goat	FRA-28	4135		metapodial	FF1:41D	-19.8	5.9			
Neolithic	Sheep/goat	FRA-32	4139		radius	FF1:41D	-20.7	4.8			
(Middle?) Neolithic	Sheep/goat	FRA-34	4141	2715	mandible	H:37	-19.7	4.6	-19.9	4.9	12.3
Middle (?) Neolithic	Sheep/goat	FRA-35	4142	2716	long bone fragment	H:37	-18.1	6.8	-18.2	7.1	13.1
Middle (?) Neolithic	Tuna	FRA-36	4143		vertebrae	H:37	-15.4	7.8			

For the Mesolithic period, the three cervids (S-EVA 4118, 4119, and 4132) and the red deer (4130) display a mean δ^13^C value of -21.2‰, which is consistent with herbivores relying on a C_3_ diet. Their δ^15^N values, however, are more variable, with 4118, 4119, and 4130 showing δ^15^N values below 6‰, while 4132 displays a nitrogen value of 6.5‰. The δ^15^N value of 4132 is higher than that of the wild boar for the same period (4129, δ^15^N: 5.8‰). While the elevated δ^15^N values for the cervid might be caused by a number of factors (e.g., the age of the specimen and/or the possibility of it grazing on ^15^N-enriched soils or seaweed) [57, 58], the relatively low values displayed by the wild boar might indicate that, despite being an omnivore, this specimen fed exclusively on C_3_ terrestrial plants.

The two canids and the feline from the Mesolithic layers at Franchthi also cluster together, displaying very similar δ^13^C (between -19.5 and -19.0‰) and δ^15^N values (between 7.4 and 7.9‰), which are consistent with a diet enriched in terrestrial animal protein expected for these animals. Lastly, the only specimen of tuna available for the Mesolithic shows the highest carbon and δ^15^N values (-14.1 and 8.7‰, respectively) among the Mesolithic animals. Although a δ^15^N value of 8.7‰ is slightly depleted compared to what would be expected from a marine environment, it is consistent with the δ^15^N values observed for bluefin tuna (*T*. *thynnus*) in several isotopic studies [59–61].

The Mesolithic humans show δ^13^C values ranging from -18.4 to -22.1‰ and δ^15^N values spanning from 6.4 to 10‰. Two individuals in particular (4144 and 4148) display relatively high δ^15^N values (8.1 and 10.0‰) and both the lowest (-22.1‰) and highest (-18.4‰) δ^13^C values of all the Mesolithic samples (with the exception of the tuna). These values indicate that individual 4144 consumed significant amounts of terrestrial animal protein. For individual 4148, given the age at death of around 6–8 months, these values would be consistent with the isotopic shift observed during breastfeeding. Specifically, during the breastfeeding period, infants typically exhibit an increase of approximately 1‰ in δ^13^C and 2–3‰ in δ^15^N compared to maternal values [62, 63]. This would imply that the mother of individual 4148 likely had isotopic values within the ranges observed in the other adult individuals in the sample, therefore indicating a terrestrial diet.

The Neolithic samples show a similar isotopic picture to the Mesolithic ones. Once again, the red deer shows the lowest δ^13^C and δ^15^N values of all the faunal remains. Conversely, the Neolithic sheep/goats display some variety in both their δ^13^C and δ^15^N values, with specimen 4142 having the highest δ^13^C (-18.1‰) and δ^15^N (6.8‰) values of all the ovicaprids. These values suggest that this animal possibly grazed on grasses growing on ^15^N-enriched soils [58]. More likely, however, we here hypothesize that this specimen either foddered on crops grown using seaweed—a practice observed for Neolithic sheep in Scotland as well [57, 64]—or was intentionally brought to graze directly on seaweed on the shorefront.

The presence of edible red, green, and brown seaweeds is well-attested in Greece, with comprehensive reviews of the different genera and species present today in the coastal areas of Aegean Sea published by Tsiamis and colleagues [65–67]. While it cannot be definitively determined which specific edible seaweed species were present during the Neolithic at Franchthi, the current abundance and diversity of edible seaweeds in the South Aegean make it plausible to assume that (at least some of) these species were also available in the past and possibly utilized as a soil fertilizer and/or as a food resource by both animals and humans.

The Neolithic pig and the canid show similar carbon (-19.8 and -19.2‰, respectively) and nitrogen (7.2 and 7.8‰, respectively) values, which are indicative of an omnivorous diet where the contribution of terrestrial animal protein was significant. In fact, it has been extensively proven [40] that the dogs at Franchthi fed on food scraps of animals left behind by humans, especially during the Neolithic. Based on the SIA results, this seems to have been true for the pigs as well. Lastly, the tuna from the Neolithic layers shows similar values to the one from the Mesolithic.

The Neolithic humans show some variability both in their δ^13^C and δ^15^N values. Individual 4151 has the lowest δ^13^C values (-22.8‰) but a δ^15^N value similar to those of the dog and the pig, which suggests that this individual consumed a mixed diet consisting of C_3_ plants and a significant amount of terrestrial animal protein. Two individuals (4153 and 4155) plot together with the canid and the pig as well, having higher δ^13^C values than 4151. This is an indication that these individuals relied intensively on the consumption of terrestrial animal protein but, unlike 4151, possibly consumed some marine resources as well. Lastly, individuals 4150 and 4154 show the highest δ^15^N values (9.7 and 8.7‰, respectively). Their δ^13^C values, however, are in the range of the values observed for the herbivores/omnivores for the same period—and are similar to the δ^13^C values observed for 4148 (Mesolithic human). We therefore argue that the isotope values for these two individuals reflect either the consumption of animals with nitrogen-enriched values (e.g., some of the ovicaprids) or of small quantities of marine resources.

Overall, the bulk isotope results suggest the Lower Mesolithic and Middle Neolithic humans from Franchthi here analyzed had a diet consisting primarily of terrestrial resources, with a few cases possibly pointing towards a small or occasional contribution from marine foods. To further examine the diet of the Lower Mesolithic and Middle Neolithic individuals from Franchthi Cave and to investigate whether—and to what extent—the contribution of marine resources into the humans’ diet is supported by amino acid data, we conducted CSIA-AA, which provides a comprehensive and high-resolution evaluation of the relative dietary contribution to tissue isotopic values [68, 69].

### Radiocarbon dates

Radiocarbon analysis results (Table 3) revealed that of the five humans, two (SSFU 2705 and 2706) are from burials that belong to the Lower Mesolithic (LM) period and the three others (SSFU 2707, 2708, and 2709) from burials from the Middle Neolithic (MN). The LM dates are compatible with previous radiocarbon datings of this phase, which has yielded calibrated dates of 8700–8300 B.C. (1σ) or 9120–8230 B.C. (2σ) [70]. No radiocarbon dates are available for the faunal remains considered in this study, but these samples have been attributed broadly to the Mesolithic and Neolithic based on their stratigraphic position at the time of their excavation. Radiocarbon dates were calibrated using the Reimer et al. calibration curve [71].

**Table 3:** Radiocarbon dates for the five Franchthi humans.

SSFU	S-EVA	Lab pretreatment no.	Franchthi number	Period	Used (mg)	Yield (mg)	% collagen	AMS code	^14^C age (BP)	1σ err.	From-to Cal BC (1σ)	From-to Cal BC (2σ;)	δ^13^C (SFU)	δ^15^N (SFU)
2705	4145	BRA 5323	Fr2	Lower Mesolithic	639	14,8	2,3	MAMS 52044	9328	28	8626–8554	8702–8477	-19.6	7.3
2706	4148	BRA 5324	Fr6	Lower Mesolithic	532	30	5,6	MAMS 52045	9294	27	8616–8482	8630–8430	-18.6	10.2
2707	4150	BRA 5325	Fr11	Middle Neolithic	501	11,5	2,3	MAMS 52046	7411	25	6362–6235	6377–6229	-19.3	10.0
2708	4154	BRA 5326	Fr37	Middle Neolithic	549	17,4	3,2	MAMS 52047	7001	24	5971–5844	5981–5802	-18.8	8.7
2709	4155	BRA 5327	Fr12	Middle Neolithic	604	14	2,3	MAMS 52048	7694	26	6569–6473	6592–6467	-20.0	6.9

### CSIA-AA

#### Quality controls

**Fig 3:**
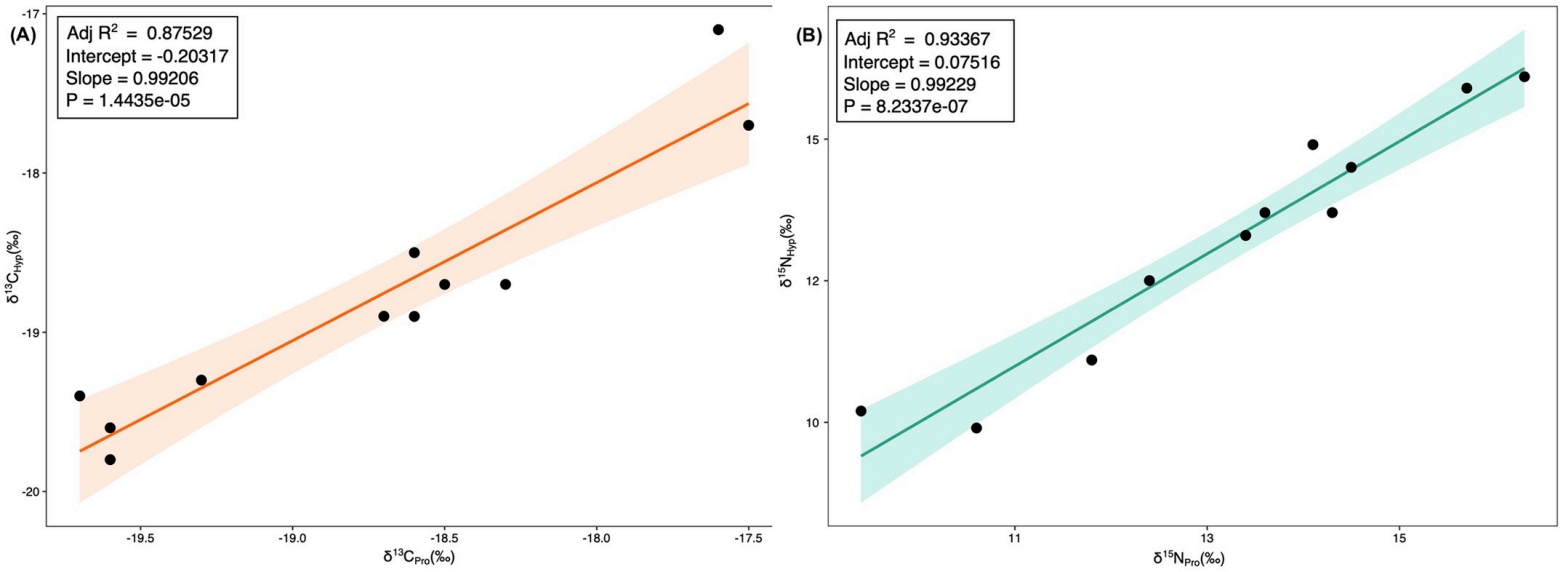
A) δ^13^C and (B) δ^15^N values of proline vs. hydroxyproline for quality controls.

By plotting the δ^13^C and δ^15^N values of proline (Pro) against hydroxyproline (Hyp), amino acid data can be quality checked to ensure that collagen was sufficiently well-preserved, and that the derivatization procedure was successful, as was instrument performance [69, 72–74]. This is because the post-translational modification of Pro into Hyp, in which the carbon-hydrogen (C-H) bond oxidizes into a carbon-hydroxyl (C-OH) bond, does not cause a C or N atom exchange. Therefore, the C and N values of both these AAs should be the same (i.e., the expected regression line for the Hyp values as a function of the Pro values should be close to *x = y*, or *R*^*2*^ *= 1*). As can be observed in Fig 3, the samples from Franchthi Cave analyzed for this study show proportional δ^13^C_Pro-Hyp_ and δ^15^N_Pro-Hyp_ correlations, with R^2^ values of 0.87 and 0.93 and p-values of 1.4435x10^-5^ and p = 8.2337x10^-7^ respectively, thus indicating good quality of the amino acid data.

Isotopic measurements of a quality control mix (QCmix) and the three internal collagen quality control (QC) standards were used for the evaluation of the quality of both the derivatization step and the instrumental analysis. Isotope results for long-term measurements of the carbon and δ^15^N values of the QC standards and QCmix are presented in S3 and S4 Tables.

#### δ^13^C_AA_

The study of the carbon isotope compositions of amino acids (AA) relies on the distinction between essential and non-essential amino acids. In humans, essential amino acids (AA_ESS_) cannot be synthesized by the body and are therefore assimilated via direct routing from dietary protein sources with little to no isotopic discrimination between consumers and their diet (i.e, Δ^13^C_consumer-diet_ ≈ 0‰) [75–79]. Hence, AA_ESS_ provide a fingerprint of the carbon isotopic variability at the base of the food chain [80–83]. Conversely, non-essential amino (AA_NESS_) are produced by the body via *de novo* synthesis reactions from AA_ESS_ or protein, carbohydrates, and fats (PCF), which cause significant isotopic discrimination between consumers and their diet (Δ^13^C_consumer-diet_ ≠ 0‰).

The δ^13^C_AA_ values obtained for the humans and fauna from Franchthi Cave (S1 Table) have been here compared to those published by Honch et al. [84], which we used as “control groups” for the four major dietary groups (C_3_, C_4_, high marine protein and high freshwater protein consumers). It has been shown [72, 84] that valine (Val) and phenylalanine (Phe), two AA_ESS_, are effective in discriminating between high marine protein (HMP) and high freshwater protein (HFP) consumers versus consumers whose diets rely on terrestrial plants. This is because, like all AA_ESS_,_,_ Val and Phe cannot be synthesized by the body and are therefore absorbed via direct routing by the organism through the consumption of dietary protein and undergo very little isotopic fractionation between the diet and the bone collagen of a consumer [75, 77]. Thus, they are considered to be reflecting the isotopic variability at the base of the food chain [80–83].

Webb and colleagues [83] have suggested that the low δ^13^C_Phe_ values observed in HFP consumers may result from the presence of terrestrial carbon (which tends to have a ^13^C-depleted signature compared to marine carbon) in the carbon pool of rivers and lakes. In terms of Val, HMP consumers usually display high δ^13^C_Val_ values, while terrestrial consumers tend to have low δ^13^C_Val_ values [84]. The δ^13^C_Phe_ vs. δ^13^C_Val_ proxy allows for the distinction between the four major dietary groups, with terrestrial plant consumers falling on a *x = y* line (R^2^ = 1), and high aquatic protein (HFP and HMP) consumers generally plotting below this line [72, 84].

**Fig 4:**
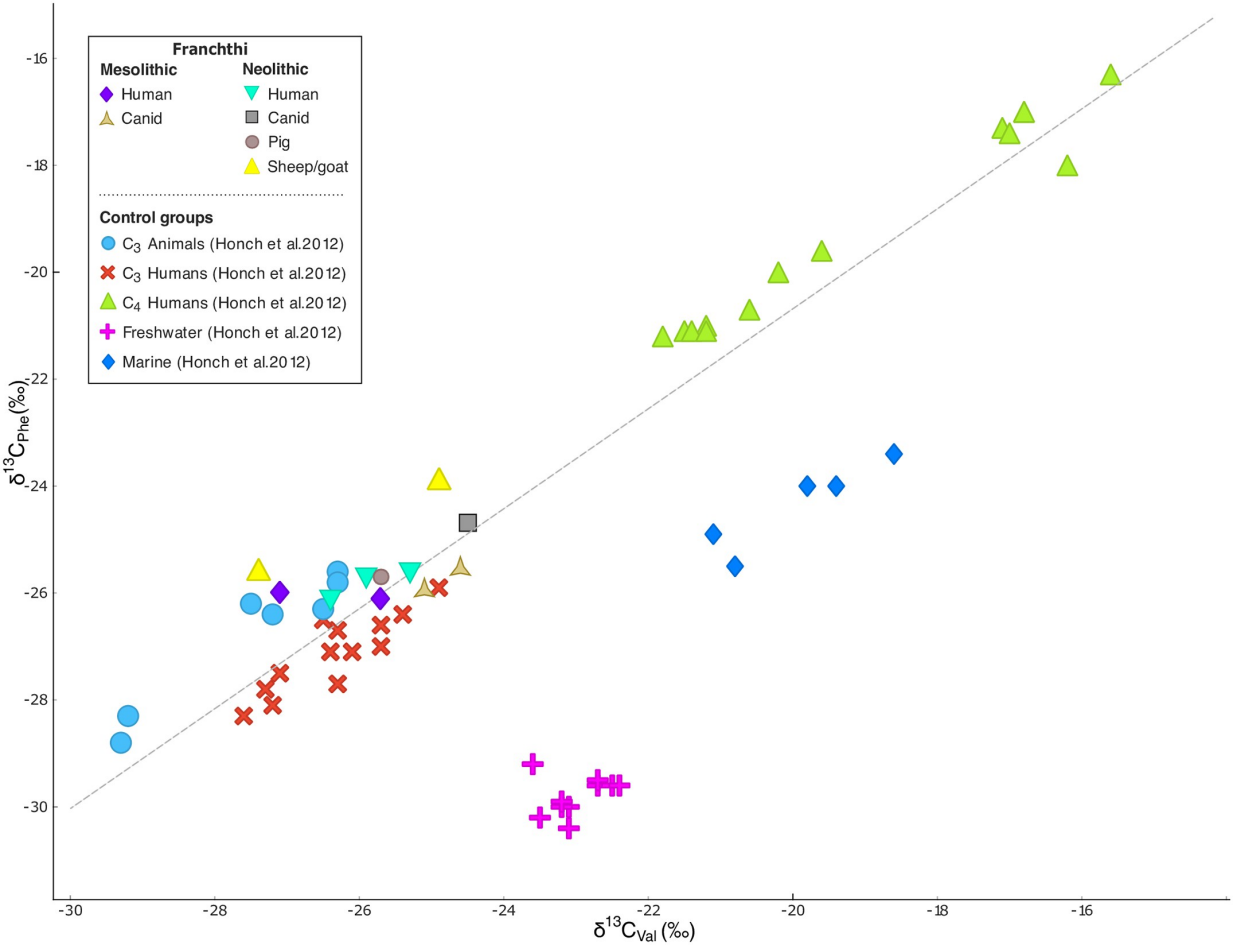
δ^13^C_Phe_ vs. δ^13^C_Val_ bivariate plot for the Franchthi samples.

As shown in the δ^13^C_Phe_ vs. δ^13^C_Val_ bivariate plot (Fig 4), all the human and animal samples from Franchthi plot on the *x = y* axis, together with the C_3_ consumers. In fact, for both humans and animals, there is little variation in the range of δ^13^C_Phe_ and δ^13^C_Val_ values (δ^13^C_Phe_ values ranging from -26.1 to -23.9‰ and δ^13^C_Val_ values from -27.4 to -24.5‰). These results suggest that they all relied on a terrestrial-based diet, mainly of C_3_ plants, while the intake of aquatic resources must have been very limited or completely absent.

**Fig 5:**
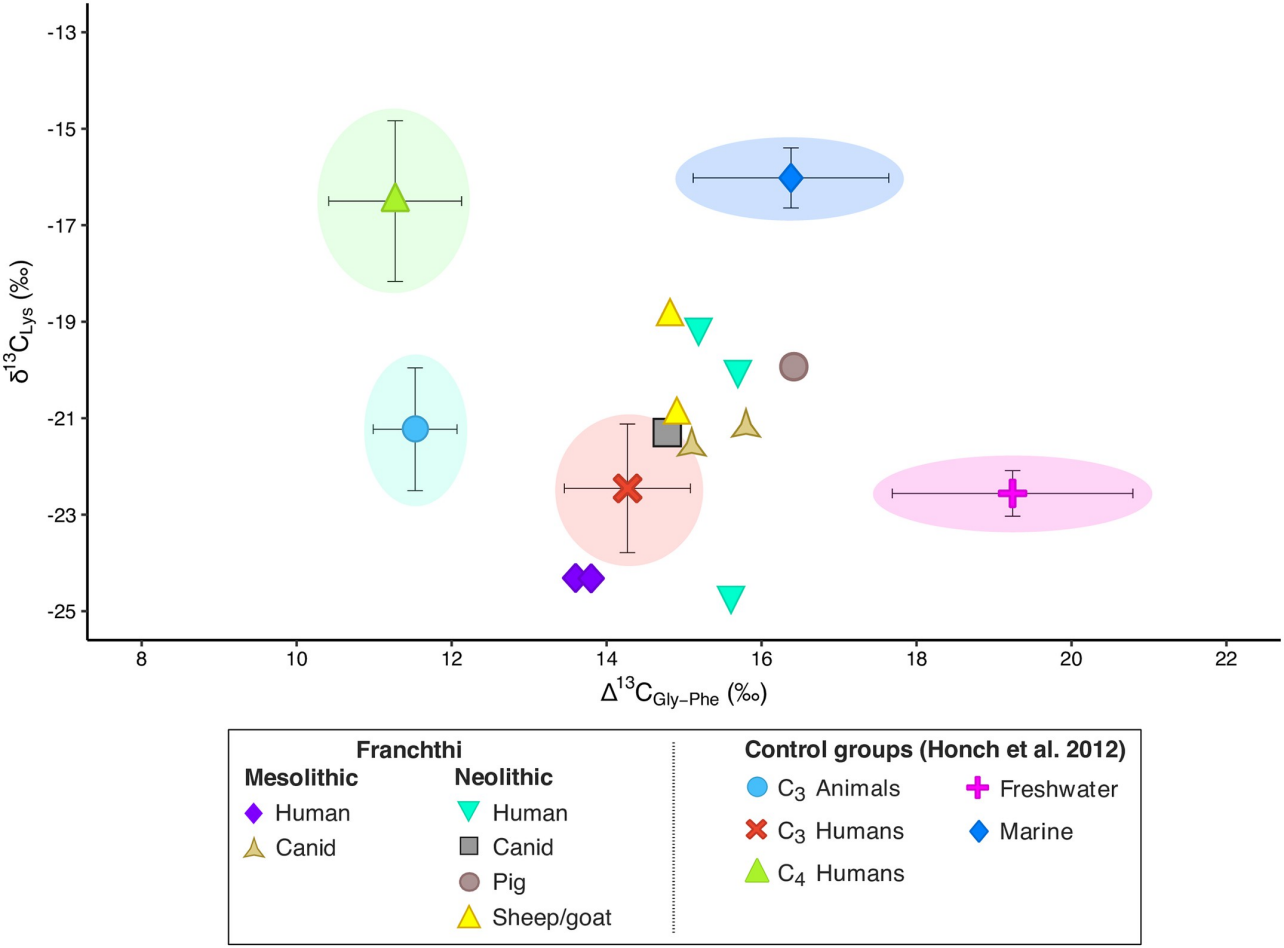
Δ^13^C_Gly–Phe_ vs. δ^13^C_Lys_ bivariate plot for the individuals from Franchthi.

We then plotted the results using the Δ^13^C_Gly−Phe_ vs. δ^13^C_Lys_ bivariate plot to try to get a better insight into the diet of the Franchthi samples (Fig 5). In archaeology, the Δ^13^C_Gly−Phe_ proxy has been proven to be useful for detecting high levels of marine protein consumption versus terrestrial diets [83–86]. In fact, since Phe is an AA_ESS_, it reflects the isotopic composition at the base of the food web.

On the contrary, marine foods tend to display higher glycine (Gly, a AA_NESS_) δ^13^C values than terrestrial food sources [84, 86] on account of the fact that marine ecosystems contain a greater number of trophic levels than the terrestrial ones [86]. Specifically, it has been suggested [83] that at high levels of marine protein consumption the Δ^13^C_Gly−Phe_ values should be relatively high (> ~15‰), whereas at high levels of terrestrial animal protein consumption, Δ^13^C_Gly−Phe_ values should be lower.

To more accurately discriminate among the dietary groups, however, the Δ^13^C_Gly−Phe_ can be plotted against the δ^13^C values of lysine (Lys, AA_ESS_) [72, 83]. Lys is commonly found in legumes, animal products, and aquatic fauna, while non-leguminous plants, including cereals, contain only very small quantities of this AA [83]. The Δ^13^C_Gly−Phe_ vs. δ^13^C_Lys_ bivariate plot for the Franchthi samples places the humans and the animals from both the Neolithic and the Mesolithic within the realm of terrestrial consumers, but with some scattering. Both the Mesolithic and the Neolithic canids analyzed here plot together close to the C_3_ consumers reference group (mean Δ^13^C_Gly−Phe_: 15.3‰; mean δ^13^C_Lys_: -21.4‰). The two Neolithic sheep/goats display very similar Δ^13^C_Gly−Phe_ values (14.9 and 14.8‰) but different δ^13^C_Lys_ values, with SSFU 2716 having a δ^13^C_Lys_ value more enriched than SSFU 2715 of ~2‰. This could be interpreted as the result of SSFU 2716 feeding on seaweed. Lastly, the Neolithic pig has the highest Δ^13^C_Gly−Phe_ values of all the samples (16.4‰) and a δ^13^C_Lys_ value of -19.9‰—in between the two Neolithic sheep.

By focusing specifically on the humans, it can be observed how the two LM individuals display slightly lower Δ^13^C_Gly−Phe_ values (13.6 and 13.8‰, respectively) compared to the three Neolithic individuals, who have Δ^13^C_Gly−Phe_ values comprised between 15.2 ad 15.7‰. These values overlap with the Δ^13^C_Gly−Phe_ values observed for both the C_3_ and the HMP and HFP consumers from our control groups, suggesting some possible intake of marine resources. The Lys values for the humans are also ambiguous as they present a stark separation between the two LM and one MN individual (SSFU 2707) on one hand (showing lower δ^13^C_Lys_ values), and the other two MN individuals (SSFU 2708 and 2709) on the other, with enriched δ^13^C_Lys_ values.

To make sense of these results, it must be noted that as a non-essential amino acid Gly may be incorporated in bone collagen either directly from dietary sources or be derived from *de novo* biosynthesis [86]. Although the body can synthesize Gly in these two major ways, most of the Gly found in collagen comes from serine (Ser), which is derived from phosphoglycerate, a glycolytic intermediate derived from glucose [86]. Accordingly, if Gly in bone collagen is mainly *de novo* synthesized, its δ^13^C values are likely to reflect the carbohydrate content of the diet, rather than that of aquatic protein.

If interpreted in this way, the highly enriched Δ^13^C_Gly−Phe_ values displayed by the Neolithic humans could be explained by the consumption of a mixed diet consisting mainly of C_3_ (possibly seaweed, as they plot in close proximity to the sheep) and likely notable quantities of terrestrial animal, rather than marine resources. Similarly, the high δ^13^C_Lys_ values shown by SSFU 2708 and 2709 could be explained with a significant consumption of terrestrial animal resources rather than fish. We here argue that the low δ^13^C_Lys_ values displayed by SSFU 2705, 2706, and 2707 are consistent with a diet consisting predominantly of terrestrial resources.

**Fig 6:**
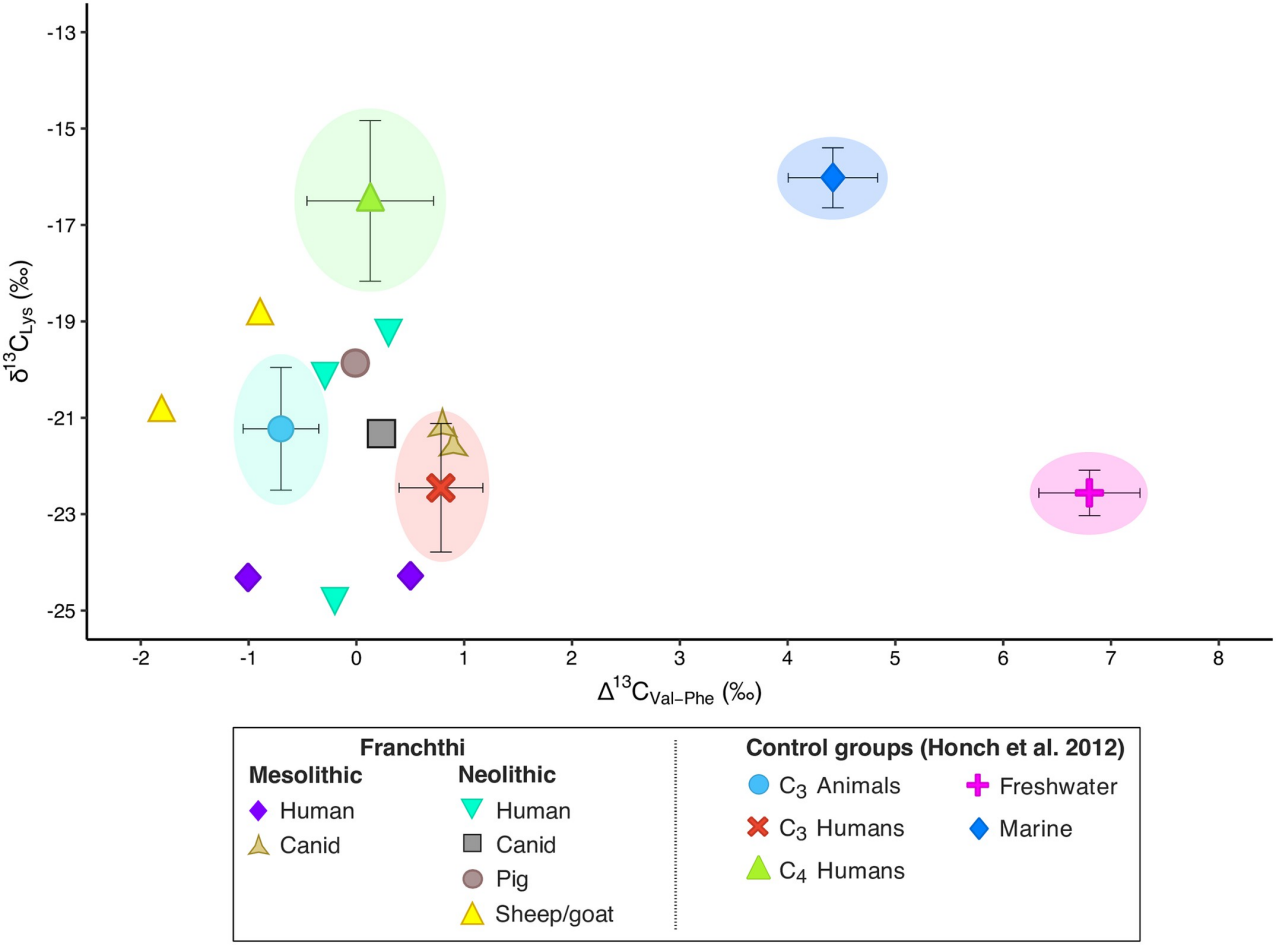
Δ^13^C_Val–Phe_ vs. δ^13^C_Lys_ bivariate plot for the individuals from Franchthi.

This hypothesis finds a strong corroboration when using the Δ^13^C_Val−Phe_ vs. δ^13^C_Lys_ proxy [83, 84] (Fig 6). Once again, the samples from Franchthi clearly plot together with the C_3_ consumers from the control groups, showing Δ^13^C_Val−Phe_ values < 1.5‰. By using this proxy, it also becomes more evident how the high δ^13^C_Lys_ values displayed by two MN humans (SSFU 2708 and 2709) is likely caused by the regular consumption of protein-enriched meats and possibly the limited consumption of marine resources (e.g., seaweed, shellfish, or fish), as these two individuals show higher Lys values than the other samples, plotting closer to the C_4_ consumers control group than the other humans. While the consumption of (wild) C_4_ plants has been previously hypothesized for the Neolithic individuals from Franchthi [43, 45], the current evidence for the presence of C_4_ plants at Franchthi is insufficient to support the hypothesis of significant consumption of such plants. Conversely, the consumption of seaweed (whether direct or indirect through the consumption of the meat of animals who fed on it) or other marine resources like shellfish would better align with the results.

Lastly, we used the AA_ESS_ Δ^13^C_Lys−Phe_ vs. Δ^13^C_Val−Phe_ marker [87] to discriminate between aquatic and terrestrial consumers. As a matter of fact, due to their inability to be synthesized by animals, AA_ESS_ obtain their carbon exclusively from dietary protein without significant modification, thus reflecting the stable carbon isotopic composition of primary production sources [87]. The Δ^13^C_Lys−Phe_ vs. Δ^13^C_Val−Phe_ proxy therefore provides the clearest separation between aquatic and terrestrial consumers, with marine and freshwater consumers having higher levels of Δ^13^C_Lys−Phe_ vs. Δ^13^C_Val−Phe_ and the terrestrial consumers displaying lower Δ^13^C_Lys−Phe_ vs. Δ^13^C_Val−Phe_ levels instead—with no overlap between the different dietary groups [87].

**Fig 7:**
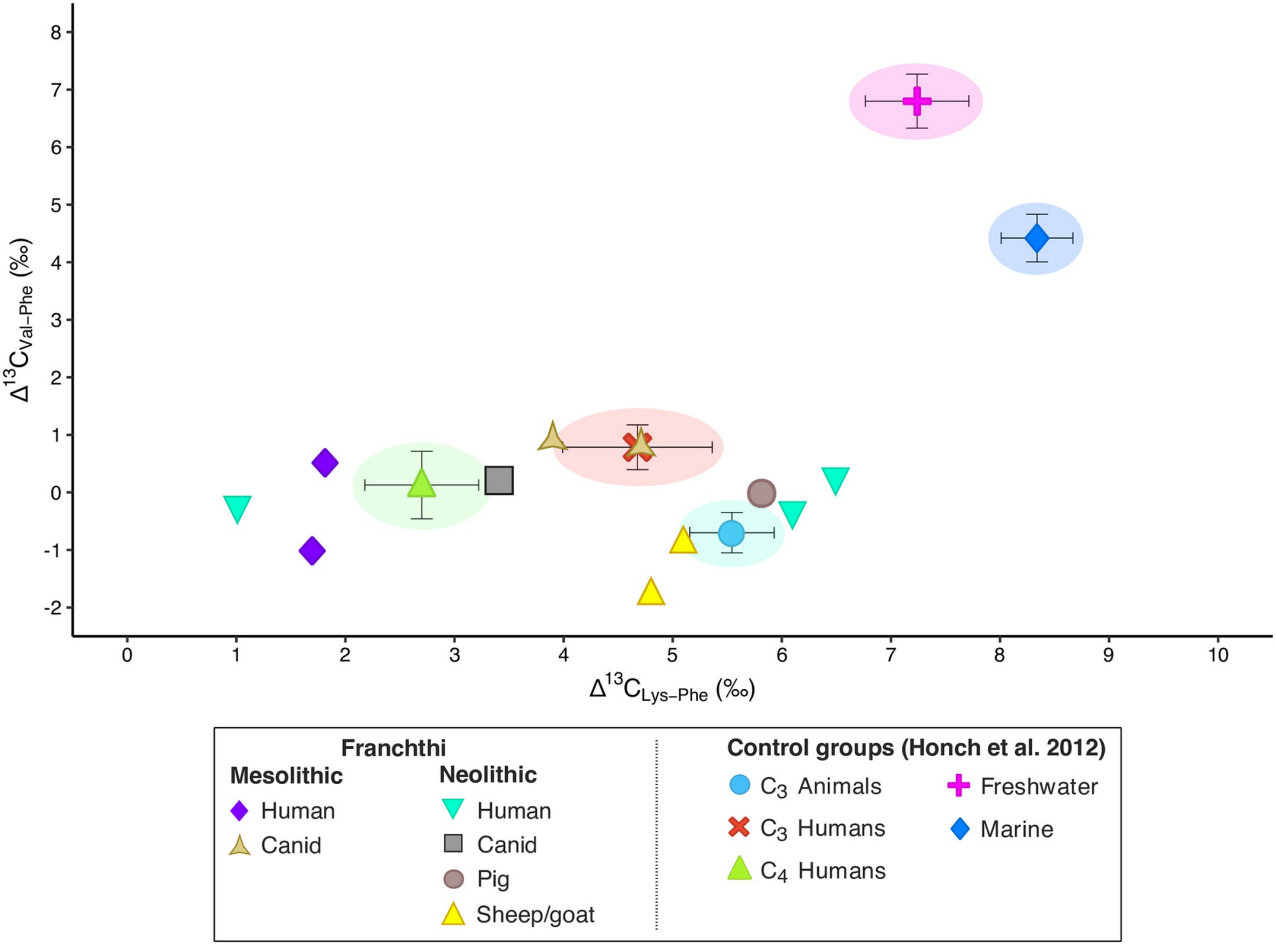
Δ^13^C_Lys–Phe_ vs. Δ^13^C_Val–Phe_ proxy for the individuals from Franchthi.

The Δ^13^C_Lys−Phe_ vs. Δ^13^C_Val−Phe_ bivariate plot for the Franchthi humans and animals (Fig 7) places both the LM and the MN individuals among the terrestrial consumers, confirming once again that their consumption of marine fauna was likely limited if not completely absent. Specifically, the two Mesolithic humans and one Neolithic human (SSFU 2707) plot together with the C_4_ consumers, while the remaining two Neolithic individuals (SSFU 2708 and 2709) clearly plot with the C_3_ consumers, although slightly towards the HMP and the HFP consumers.

As previously mentioned, the substantial consumption of wild C_4_ plants at Franchthi lacks support from the archaeobotanical record of either the Mesolithic or the Neolithic period. In this context, we are inclined to interpret the findings for the two LM and one MN individuals as indicative of terrestrial animal resource consumption. Regarding the other two Neolithic individuals, the patterns observed in the Δ^13^C_Lys−Phe_ vs. Δ^13^C_Val−Phe_ values further suggest the possibility of (direct or indirect) seaweed or small quantities of low trophic marine resources (e.g., shellfish) consumption.

#### δ^15^N_AA_

Although the application of different δ^13^C_AA_ proxies is useful to distinguish between C_3_, C_4_, HFM and HFP diets, the δ^13^C values of individual amino acids do not provide a clear indication on the level of protein consumption in the diet [88]. However, it is possible to determine a consumer’s trophic position (TP) at a high resolution by comparing the *δ*^15^N values of trophic (AA_Tr_) vs. source (AA_Src_) amino acids [79, 89]. The distinction between AA_Tr_ and AA_Src_ is based on whether their retain (AA_Src_) or lose (AA_Tr_) nitrogen atoms during metabolic reactions such as deamination and transamination [46, 90, 91].

**Fig 8:**
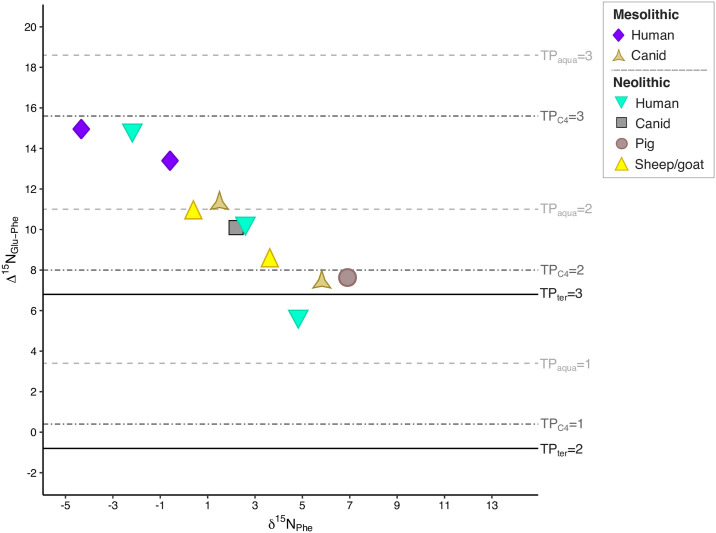
Δ^15^N_Glu-Phe_ vs. δ^15^N_Phe_ bivariate plot for the Franchthi samples. TP estimations based on the equation and data from: [89, 92].

The δ^15^N_AA_ results from Franchthi are illustrated in S2 Table. The Δ^15^N_Glu-Phe_ vs. δ^15^N_Phe_ bivariate plot for the Franchthi samples (Fig 8) confirms the original hypothesis that the analyzed humans from this cave had a diet consisting of predominantly of terrestrial resources, with limited intake of marine resources. The two Mesolithic and one Neolithic individuals (SSFU 2707) have TPs of 4.1, 3.9, and 4.1, respectively. While these individuals position well above the TP for terrestrial carnivores and towards TP_aqua_ = 3 (thus suggesting the consumption of marine resources), we must also take into consideration the results from carbon amino acid proxies, which consistently place these individuals in the realm of terrestrial consumers.

Therefore, we interpret the elevated Δ^15^N_Glu-Phe_ values displayed by the two Lower Mesolithic and one Middle Neolithic individual to reflect the significant consumption of terrestrial meat enriched in δ^15^N, rather than marine resources. This hypothesis would be corroborated, especially for the MN individual, by the particularly high Δ^15^N_Glu-Phe_ values of both Neolithic sheep/goats analyzed here, which place these herbivores above the TP for terrestrial carnivores and therefore strongly suggest that they did, in fact, graze on seaweed. Lastly, SSFU 2708 places above TP_ter_ = 3, indicating a mixed diet where the contribution of terrestrial animal protein was significant, but likely came from different and more varied sources than for SSFU 2707, while SSFU 2709 plots just below TP_ter_ = 3. For this last individual, suggesting a much lower consumption of terrestrial animal protein than the other individuals.

The determination of TDF’s based on δ^15^N_Glu_ has, however, been challenged [82, 93, 94] for being highly variable and influenced by a number of factors, such as species, tissue type, excretion mode, diet quality and quantity, and pathophysiologic conditions. The δ^15^N values of threonine (Thr) have been recently investigated as indicators of high protein intake [68, 95]. In mammals, high dietary protein levels have been found to increase the activity of the enzyme threonine ammonia-lyase, which causes a depletion in the δ^15^N values of Thr. Consequently, herbivores tend to display significantly higher levels of δ^15^N_Thr_ than high protein consumers [96].

As can be observed in Table 4, the Neolithic dog from Franchthi shows the lowest δ^15^N_Thr_ values (-18.2‰). Similarly low δ^15^N_Thr_ values are displayed by the two Mesolithic dogs (-15.5 and -15.1‰ respectively). These results are consistent with the carnivorous/omnivorous diet expected from canids. On the contrary, the two Neolithic sheep/goats display the highest δ^15^N_Thr_ values (-6.6 and -4.0‰), which agrees with their exclusively herbivorous diet. Therefore, their elevated Δ^15^N_Glu-Phe_ values may indeed have been influenced by the consumption of seaweed. Interestingly, the Neolithic pig shows relatively a high Thr nitrogen value (-10.7‰), which indicates that, despite being an omnivorous animal, it was mostly fed on low-protein foods, such as cereals or nuts.

**Table 4:** δ^15^N_Thr_ values for the Franchthi samples.

SSFU	Sample	Threonine (‰)
2705	Human Lower Mesolithic	-11.1
2706	Human Lower Mesolithic	-17.5
2707	Human Middle Neolithic	-11.7
2708	Human Middle Neolithic	-12.0
2709	Human Middle Neolithic	-9.3
2710	Canid Mesolithic	-15.5
2711	Canid Mesolithic	-15.1
2713	Canid Neolithic	-18.2
2714	Pig Neolithic	-10.7
2715	Sheep/goat Neolithic	-6.6
2716	Sheep/goat Neolithic	-4.0

The δ^15^N_Thr_ values for the Franchthi humans are particularly interesting. SSFU 2706 (LM) displays the lowest δ^15^N_Thr_ levels of all humans (-17.5‰), clearly indicating that this individual relied heavily on the consumption of terrestrial products particularly rich in protein. Considering that this individual was an infant aged 6–8 months, its low Thr values may be attributed to breastfeeding. The other LM individual displays a δ^15^N_Thr_ value of -11.1‰, reflecting a more moderate protein-based diet than what was suggested by the Δ^15^N_Glu-Phe_ vs. δ^15^N_Phe_ proxy.

As for the Neolithic individuals, SSFU 2709 shows the highest δ^15^N_Thr_ values, which, as already suggested by the Δ^15^N_Glu-Phe_ vs. δ^15^N_Phe_ proxy, points towards a diet where the intake of terrestrial animal protein must have been limited. The δ^15^N_Thr_ values of SSFU 2707 and 2708 are consistent with a high-protein terrestrial diet [96].

To further support our hypotheses, we conducted principal component analysis (PCA) on selected human and faunal remains from Franchthi cave using PAST4 [97]. Detailed information about the PCA analysis is provided in S8 Table. Recently, Choy and colleagues [98] explored different combinations of essential and source amino acids to discern the most efficient in discriminating between various food sources in their study on Korean prehistoric communities. We adopted a slightly modified version of their combination, considering four essential amino acids (Thr, Val, Leu, Phe) and three source amino acids (Val, Leu, Phe). Unfortunately, our study was conducted before the publication of Choy and colleagues’, and Ile was not measured. Despite this limitation we still achieved a good degree of separation of the different dietary groups, with PC1 (49.62% of variance) separating terrestrial vs marine foods and PC2 (26.43% of variance) discriminating between C_3_ and C_4_ plants.

**Fig 9:**
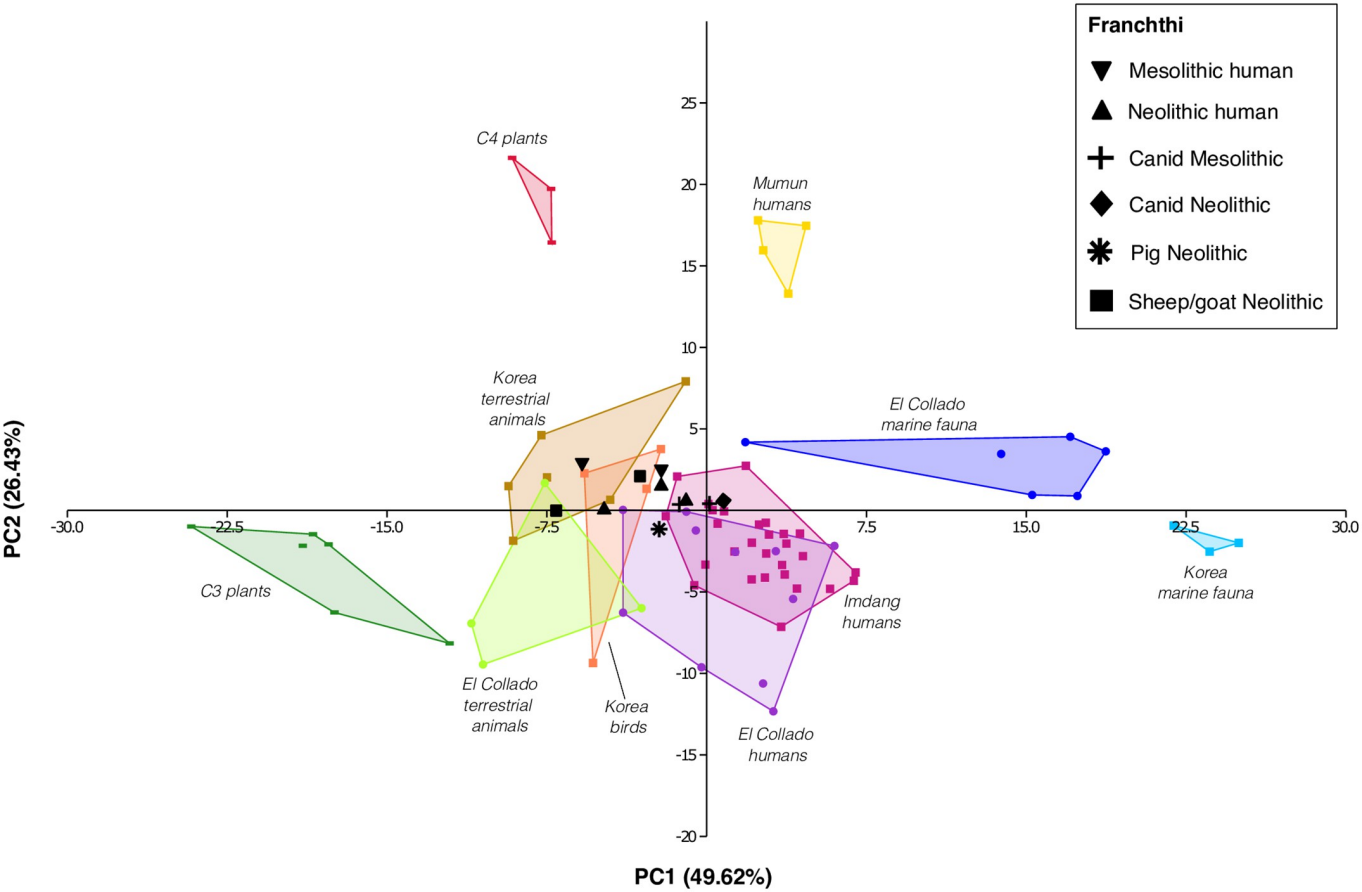
PCA for the humans and associated fauna from Franchthi. Data for comparison from Choy et al. (2024) and Fontanals-Coll et al. (2023).

In our analysis, we included the data from Choy and colleagues to ensure that our PCA, while excluding Ile, remained consistent with their findings. We also considered the CSIA-AA results obtained by Fontanals-Coll and colleagues [18] from El Collado (Spain), another Mesolithic coastal site in the Mediterranean. As can be seen in Fig 9, even without Ile, the PCA confirms the results obtained by Choy et al. and Fontanals-Coll et al. in showing a wide range of dietary strategies for the prehistoric humans from Korea and Spain. Specifically, the Mumun individuals (yellow squares) still cluster in the realm of C_4_ consumers, while both the Imdang (hot pink squares) and the El Collado humans (purple dots) show a wide range of subsistence strategies including the exploitation of marine foods, as the authors concluded in their respective studies. However, the humans from Franchthi clearly plot in the realm of terrestrial consumers, together with the terrestrial herbivores from the reference groups, confirming the δ^13^C_AA_ and δ^15^N_AA_ proxy results that suggest they likely did not rely on marine resources for their diet.

Lastly, Table 5 provides a comprehensive summary of our findings for the Lower Mesolithic and Middle Neolithic individuals analyzed in this paper based on the various amino acid proxies and the principal component analysis (PCA).

**Table 5:** Summary of the CSIA proxy results for the Franchthi humans.

Proxy/Sample	δ^13^C_Phe_ vs. δ^13^C_Val_	Δ^13^C_Gly−Phe_ vs. δ^13^C_Lys_	Δ^13^C_Val−Phe_ vs. δ^13^C_Lys_	Δ^13^C_Lys−Phe_ vs. Δ^13^C_Val−Phe_	Δ^15^N_Glu-Phe_ vs. δ^15^N_Phe_	δ^15^N_Thr_	PCA
**2705 (LM)**	Terrestrial diet	Terrestrial diet with possible intake of marine resources	Terrestrial diet	Terrestrial Diet	Terrestrial diet (high protein consumption)	Terrestrial diet (high protein consumption)	Terrestrial diet
**2706 (LM)**	Terrestrial diet	Terrestrial diet with possible intake of marine resources	Terrestrial diet	Terrestrial Diet	Terrestrial diet (high protein consumption)	Terrestrial diet (high protein consumption) [breastfeeding?]	Terrestrial diet
**2707 (MN)**	Terrestrial diet	Terrestrial diet	Terrestrial diet	Terrestrial diet	Terrestrial diet (high protein consumption)	Terrestrial diet (high protein consumption)	Terrestrial diet
**2708 (MN)**	Terrestrial diet	Terrestrial diet	Terrestrial diet with possible, limited intake of marine resources	Terrestrial diet with possible, limited intake of marine resources	Terrestrial diet	Terrestrial diet (high protein consumption)	Terrestrial diet
**2709 (MN)**	Terrestrial diet	Terrestrial diet	Terrestrial diet with possible, limited intake of marine resources	Terrestrial diet with possible, limited intake of marine resources	Terrestrial diet	Terrestrial diet	Terrestrial diet

## Conclusions

Franchthi Cave, located in the southwestern Peloponnese, is one of the few sites in Greece to present a stratigraphic sequence that ranges from the Upper Paleolithic through the Final Neolithic. Franchthi’s rich stratigraphic sequence makes it an optimal site for investigating shifts in subsistence strategies during pivotal transitional periods, such as the Mesolithic to Neolithic transition in the Mediterranean. Unlike other regions in Europe, where Mesolithic hunter-gatherer communities primarily relied on pelagic resources, the Mediterranean’s distinctive biogeographical qualities seem to have limited such sustenance options. As a result, investigating subsistence patterns at Franchthi provides a valuable lens into the subsistence strategies of the communities that frequented the cave before and after the arrival of the “Neolithic package” to the region.

In this paper, we presented new results from δ^13^C and δ^15^N bulk collagen stable isotope analysis, ^14^C dates and compound-specific stable isotope analysis of individual amino acids for five humans and six animals from the Lower Mesolithic and Middle Neolithic at Franchthi Cave. Our results confirm that the analyzed humans from selected periods in the Mesolithic and Neolithic at Franchthi consumed a terrestrial diet primarily based on the consumption of animal products. Our results do not indicate that the Franchthi individuals here analyzed consumed significant amounts of marine resources, although we do not exclude the occasional consumption of fish and marine molluscs, especially in the absence of amino acid data for these resources. Despite the numerous remains of shallow-water fish and sea shells, however, the consumption of such resources during the Lower Mesolithic was not significant enough to leave a distinct isotopic signature.

Our isotope results for the Middle Neolithic reveal that sheep were likely grazing on the shore (possibly on seaweed), and that humans relied on a diet consisting primarily of terrestrial animal protein—mostly meat and milk deriving from the sheep that were grazing on the shore—and/or possibly on the direct consumption of seaweed, although this latter hypothesis is more difficult to prove due to the inability of seaweed to preserve in the archaeological record and to the lack of AA data for this resource in the context of prehistoric Greece.

In conclusion, we argue that the consumption of aquatic resources at Franchthi was at most occasional or seasonal for the individuals analyzed in this study, but not significant enough to be revealed by the amino acid data. This is in accordance with the prehistoric patterns of seasonal exploitation of pelagic resources observed at Franchthi and other Aegean sites [21, 42], as well as with the zooarchaeological record from the Lower Mesolithic layers—although for the Middle Neolithic the zooarchaeological assemblage seems to overestimate the contribution of marine resources in human diets, at least for the individuals from this time period analyzed here. However, it is important to note that we were not able to analyze samples from contexts where the density of fish bones is highest (Late Upper Paleolithic, Upper Mesolithic, and Early Late Neolithic). Thus, while our findings are significant for the Lower Mesolithic and Middle Neolithic layers specifically, they of course do not fully represent the extent of marine resource consumption at Franchthi Cave during the Mesolithic and Neolithic as a whole.

## Supporting information

S1 Table Carbon isotope results for all Franchthi samples. Isotopic values are an average of triplicate measurements, the ± values are the standard deviation of the triplicate measurements.
(XLSX)

S2 Table Nitrogen isotope results for all Franchthi samples. Isotopic values are an average of triplicate measurements, the ± values are the standard deviation of the triplicate measurements.
(XLSX)

S3 Table Carbon isotope results for collagen QC standards (SRM-1, SRM-2, and SRM-3) from the two analytical sessions in comparison with long-term average values. Isotopic values are an average of triplicate measurements, the ± values are the standard deviation of the triplicate measurements.
(XLSX)

S4 Table Nitrogen isotope results for collagen QC samples (SRM-1, SRM-2, and SRM-3) from the two analytical sessions in comparison with long-term average values. Isotopic values are an average of triplicate measurements, the ± values are the standard deviation of the triplicate measurements.
(XLSX)

S5 Table Carbon isotope results for QCmix from both analytical sessions in comparison with long-term average values. Isotopic values are an average of triplicate measurements, the ± values are the standard deviation of the triplicate measurements.
(XLSX)

S6 Table Nitrogen isotope results for QCmix from both analytical sessions in comparison with long-term average values. Isotopic values are an average of triplicate measurements, the ± values are the standard deviation of the triplicate measurements.
(XLSX)

S7 Table Samples originally analyzed for bulk collagen δ^13^C and δ^15^N isotope analysis at MP-EVA. (XLSX)

S8 Table PCA data for Franchthi Cave. (XLSX)

S1 Fig Typical CO_2_ gas chromatogram of NAIP ester derivatized amino acids from bone collagen (S-SFU 2716). Ala = alanine, Val = valine, Gly = glycine, Leu = leucine, Nle = norleucine, Pro = proline, Thr = threonine, Asx = aspartic acid, Ser = serine, Glx = glutamic acid, Phe = phenylalanine, Hyp = hydroxyproline, Lys = lysine.
(PNG)

S2 Fig Typical N_2_ gas chromatogram of NAIP ester derivatized amino acids from bone collagen (S-SFU 2707). Ala = alanine, Gly = glycine, Val = valine, Leu = leucine, Nle = norleucine, Thr = threonine, Ser = serine, Pro = proline, Asx = aspartic acid, Glx = glutamic acid, Hyp = hydroxyproline, Phe = phenylalanine, Lys = lysine.
(PNG)
