# Supplementary figures and images for: High-resolution isotope dietary analysis of Mesolithic and Neolithic humans from Franchthi Cave, Greece
Source: PLoS One. 2025 Jan 17;20(1):e0310834. doi: 10.1371/journal.pone.0310834 (PMC11741403; doi:10.1371/journal.pone.0310834)

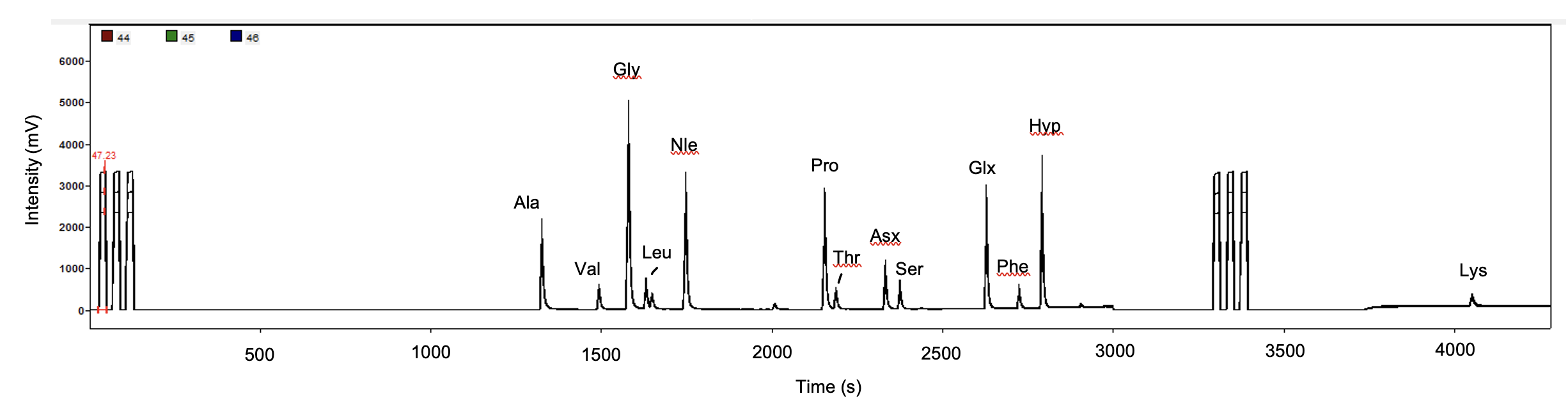

Supplement: S1 Fig — Ala = alanine, Val = valine, Gly = glycine, Leu = leucine, Nle = norleucine, Pro = proline, Thr = threonine, Asx = aspartic acid, Ser = serine, Glx = glutamic acid, Phe = phenylalanine, Hyp = hydroxyproline, Lys = lysine. (PNG) [file pone.0310834.s009.png]

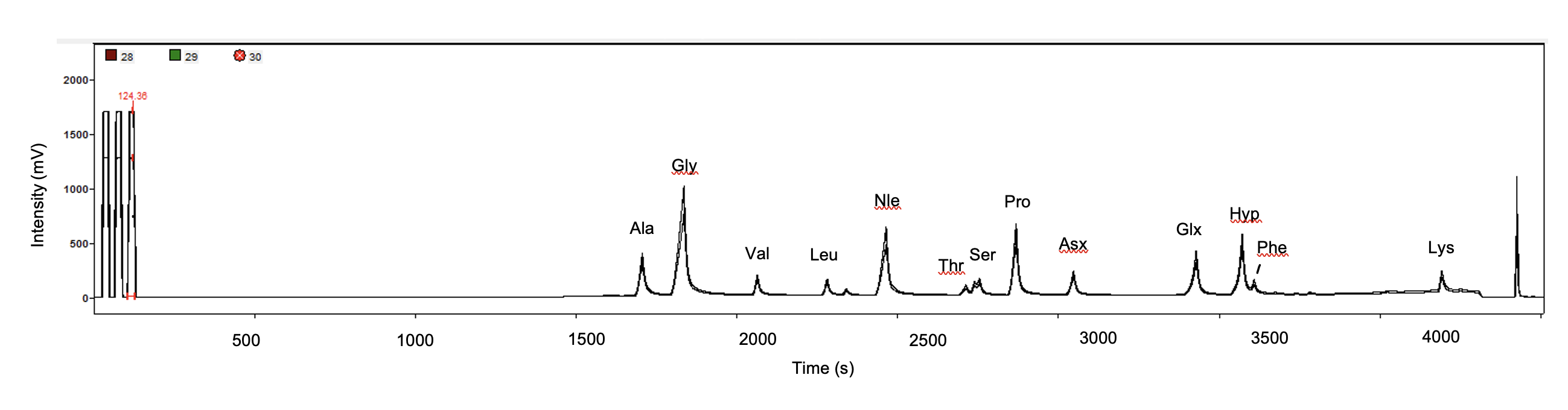

Supplement: S2 Fig — Ala = alanine, Gly = glycine, Val = valine, Leu = leucine, Nle = norleucine, Thr = threonine, Ser = serine, Pro = proline, Asx = aspartic acid, Glx = glutamic acid, Hyp = hydroxyproline, Phe = phenylalanine, Lys = lysine. (PNG) [file pone.0310834.s010.png]
